# Dosimetric Comparison of the Heart and Left Anterior Descending Artery in Patients With Left Breast Cancer Treated With Three-Dimensional Conformal and Intensity-Modulated Radiotherapy

**DOI:** 10.7759/cureus.21108

**Published:** 2022-01-11

**Authors:** Ayush Garg, Piyush Kumar

**Affiliations:** 1 Radiation Oncology, Shri Ram Murti Smarak Institute of Medical Sciences, Bareilly, IND

**Keywords:** left anterior descending artery, conformal radiotherapy, heart, dosimetric parameters, left breast radiotherapy, breast cancer

## Abstract

Background

Adjuvant radiotherapy plays an important role in the management of breast cancer, along with surgery and chemotherapy. However, postoperative radiotherapy poses an increased risk of radiation-induced heart diseases in patients with left-sided breast cancer due to damage of the coronary arteries, which can cause myocardial fibrosis and coronary artery disease; however, there is a lack of sufficient evidence for it. Hence, the present study aimed to assess the dosimetric parameters of the heart and left anterior descending (LAD) coronary artery in patients with left breast cancer treated with three-dimensional conformal radiotherapy (3DCRT) and intensity-modulated radiotherapy (IMRT).

Methodology

This study included 20 patients with left-sided breast cancer treated between January and July 2019. Patients were equally divided into two groups as follows: group I included those treated with 3DCRT and group II included those treated with IMRT. Radiotherapy was administered to the chest wall and regional lymph nodes. The dose administered for the planning target volume was 50 Gy in 25 daily fractions over five weeks with 6 MV photons. Dosimetric parameters of planning tumor volume (PTV; V95%, V90%, Dmax, Dmin, Dmean, V53.5 Gy, conformity index, and homogeneity index) along with the heart (V5%, V30%, and Dmean) and LAD artery (mean and V25%) were evaluated. Dose-volume histograms were generated and compared. The LAD artery was contoured virtually retrospectively during the study to determine the dosimetric parameters; the dose to the LAD artery was not considered during planning.

Results

Dosimetric parameters of the PTV were similar for 3DCRT and IMRT; D95 (38.53 vs. 41.61 Gy), D90 (43.67 vs. 44.77 Gy), Dmean (48.3 vs. 48.72 Gy), conformity index (1.10 vs. 1.06), and homogeneity index (0.50 and 0.28) did not show a significant difference. The Dmean for the LAD artery was significantly higher than that for the heart on 3DCRT (23.66 Gy vs. 8.46 Gy; p < 0.0000) and IMRT (31.53 vs. 17.7 Gy; p < 0.0000). The V25 for the LAD artery was significantly higher than that for the heart on 3DCRT (40.27 vs. 14.13 Gy; p < 0.0024) and IMRT (66.21 vs. 27.74 Gy; p < 0.0002).

Conclusions

Radiation doses to the LAD artery and heart must be evaluated prior to radiotherapy in patients with left breast cancer. Long-term follow-up is needed to evaluate cardiac complications and their association with dosimetric parameters.

## Introduction

Radiotherapy plays a key role in the multimodal treatment of breast cancer in most cases. However, radiation doses to the heart and lungs cannot be estimated accurately in conventional radiotherapy techniques. This leads to unintentional long-term cardiac and pulmonary toxicities. Additionally, increased incorporation of computed tomography (CT) scans in radiotherapy planning and utilization of newer conformal radiotherapy techniques have resulted in increased doses to the heart and lungs.

Women with left-sided breast cancer who undergo adjuvant radiotherapy have increased cardiac morbidity [1] and mortality [2] compared to women with right-sided breast cancer who undergo adjuvant radiotherapy. Radiation-associated heart diseases involve a spectrum of clinical diagnoses, including pancarditis, pericarditis, cardiomyopathy, and coronary heart disease with ischemic heart disease. Clinical presentations of radiation-induced heart disease have been observed in patients who received therapeutic doses of ≥35 Gy to partial volumes of the heart [3].

A landmark population-based study determined that patients with breast cancer who were treated with adjuvant radiotherapy had a 1.7-fold increased risk of cardiac death compared to those treated with surgery alone [2]. Additionally, a linear correlation between median heart dose and increased risk of major cardiac events strengthens the association between breast radiotherapy and heart damage [4]. It has been reported that, with each additional 1 Gy of mean heart dose, there is a 7.4% incremental risk of major coronary events [4].

During radiotherapy, the dose is not distributed homogeneously in the heart; the highest doses are likely to be delivered to the anterior part of the heart, including the left anterior descending (LAD) coronary artery. This is concerning because studies have suggested that arteries are sensitive to radiation, and the LAD artery is one of the typical sites of origin for ischemic heart disease [5]. Dose restriction to the entire heart to prevent pericarditis and cardiovascular mortality is well established; however, the tolerance dose for the LAD artery remains unclear [6].

This study aimed to assess the dosimetric parameters of the heart and LAD artery in patients with left breast cancer who were treated with three-dimensional conformal radiotherapy (3DCRT) and intensity-modulated radiotherapy (IMRT).

This article was previously presented as a meeting abstract at the 41st Annual Conference of the Association of Radiation Oncologists of India (A9) on November 29, 2019.

## Materials and methods

This was a single-center, retrospective, observational study. Institutional Review Board approval was not needed for this retrospective study according to our hospital’s policies. We included 20 patients with left-sided breast cancer who were treated from January to July 2019.

Inclusion and exclusion criteria

Patients who had left-sided breast cancer, had undergone a modified radical mastectomy, and were aged ≥18 years were included in this study. Patients who had undergone previous breast surgery or radiotherapy, presented with other metastatic diseases, and had undergone breast-conserving surgery were excluded.

Radiotherapy planning

A simulation using contrast-enhanced CT with a slice thickness of 3 mm was performed with the patients’ hands above their heads. Patients were equally divided into two groups: groups I and II included those who were treated with 3DCRT and IMRT, respectively.

Delineation

The delineation of clinical target volume and heart was performed as per the Radiation Therapy Oncology Group Breast Cancer Atlas [7]. The LAD artery was contoured virtually retrospectively from its origin on the left main coronary artery down to the last visible segment of the vessel using the guidelines by Lee et al. [8].

3DCRT Planning

Two tangential beams were used for chest wall irradiation with gantry angles ranging from 225° to 315° on the lateral side and 45° to 135° on the medial side. A separate field was used from a 0° gantry angle for supraclavicular nodal irradiation. The isocenter was placed at the junction of the tangential fields and supraclavicular field, and a half-beam block technique with asymmetric jaws was used. Using the beam eye view, the beam angles were chosen to optimize the coverage of the pretreatment tumor volume with the maximum exclusion of the organs at risk. The fields extended a minimum of 1 cm anteriorly from the chest wall to provide coverage of the “flash” region. Enhanced dynamic wedges and field-in-field techniques were used when required for homogeneous dose distribution.

IMRT Planning

A total of five to seven semi-opposing (mostly tangential) beams were used in IMRT plans. Inverse planning was performed, and beam optimization was used to achieve the desired objectives and constraints. Tissue inhomogeneities were considered during the beam optimization process. The optimal planning objectives for both techniques were specified as the dose to the planning tumor volume (PTV) between 95% and 107% relative to the 100% prescription point.

Dose Prescription

Radiotherapy was administered to the chest wall and regional lymph node areas. The dose administered to the planning target volume was 50 Gy in 25 daily fractions over five weeks with 6 MV photons.

Plan Evaluation

Dosimetric parameters of PTV (V95%, V90%, Dmax, Dmin, Dmean, V53.5 Gy, conformity index, and homogeneity index) along with those of the heart (V5%, V30%, and Dmean) and LAD artery (mean and V25%) were evaluated. Dose-volume histograms were generated and compared. The LAD artery was contoured virtually retrospectively to determine dosimetric parameters, and the dose to the LAD artery was not considered during planning.

Statistical analysis

Statistical significance was set at p-values of <0.05. The chi-square test was used to determine the significance of study parameters on the categorical scale between the two groups. The Student’s t-test (two-tailed, dependent) was used to determine the significance of study parameters, such as the correlation between various dosimetric parameters on a continuous scale in each group.

## Results

Dosimetric parameters of PTV were statistically and significantly better in IMRT than 3DCRT for V95% (p = 0.000) and V90% (p = 0.005). The other dosimetric parameters did not show any statistically significant differences (Table 1).

**Table 1:** PTV parameters. PTV: planning tumor volume; 3DCRT: three-dimensional conformal radiotherapy; IMRT: intensity-modulated radiotherapy; V95%: percentage volume of structure receiving 95% dose; V90%: percentage volume of structure receiving 90% dose; Dmax: maximum dose; Dmin: minimum dose; Dmean: mean dose; CI: conformity index, HI: homogeneity index

PTV parameters	3DCRT	IMRT	P-value
V95 (%)	82.24	93.69	0.000
V90 (%)	89.27	95.07	0.005
Dmax (Gy)	55.49	54.81	0.32
Dmean (Gy)	48.30	48.72	0.68
Dmin (Gy)	10.47	19.01	0.09
V53.5 Gy	4.53%	0.62%	0.07
CI	1.10	1.06	0.78
HI	0.50	0.28	0.16

The Dmean for the LAD artery was significantly higher than that of the heart for both 3DCRT (23.66 Gy vs. 8.46 Gy; p < 0.000) and IMRT (31.53 vs. 17.7 Gy; p < 0.000) (Table 2), which points that the LAD artery may be an important structure to estimate late cardiac toxicity.

**Table 2:** Comparison between mean doses of the heart and LAD artery. 3DCRT: three-dimensional conformal radiotherapy; IMRT: intensity-modulated radiotherapy; LAD: left anterior descending

	LAD artery	Heart	P-value
3DCRT	23.66 Gy	8.46 Gy	0.000
IMRT	31.53 Gy	17.70 Gy	0.000

Compared to IMRT, 3DCRT delivered a significantly lower dose (Dmean) for both the LAD artery (p = 0.02) and heart (p = 0.000); other LAD artery parameters (V25%, p = 0.014) and heart parameters (V5% and V30%, p < 0.000 and p = 0.08, respectively) were also significantly better in 3DCRT than IMRT. The maximum dose to the LAD artery was similar in 3DCRT and IMRT plans (Table 3).

**Table 3:** LAD and heart parameters. Mean: mean dose; Max: maximum dose; V5%: percentage volume of structure receiving 5% dose; V25%: percentage volume of structure receiving 25% dose; V30%: percentage volume of structure receiving 30% dose; 3DCRT: three-dimensional conformal radiotherapy; IMRT: intensity-modulated radiotherapy

	Parameters	3DCRT	IMRT	P-value
LAD artery	Mean (Gy)	23.66	31.53	0.02
	V25 (%)	40.27	66.21	0.014
	Max (Gy)	48.68	48.22	0.66
Heart	V5 (%)	23.74	82.50	<0.000
	V30 (%)	12.94	20.2	0.08
	Mean (Gy)	8.46	17.70	0.000

## Discussion

Dosimetric parameters of PTV were statistically and significantly better in IMRT than 3DCRT for V95% and V90%. The other dosimetric parameters did not show any statistically significant differences. The Dmean for the LAD artery was significantly higher than that of the heart in both 3DCRT (23.66 Gy vs 8.46 Gy; p < 0.000) and IMRT (31.53 vs. 17.7 Gy; p < 0.000).

Although there has been a considerable decrease in heart doses over the past few years [9-11], radiation-induced heart disease remains a concern due to improvement in the survival of patients with breast cancer. The mean heart dose is often used as the reference measure for cardiotoxicity studies [5]. However, there is an increasing interest in doses to individual cardiac substructures as some studies have implicated the LAD artery [12,13] and left ventricle [14] as important areas of the heart associated with radiation-induced heart disease.

Evaluating the mean or Dmax doses to the LAD artery requires precise delineation of this structure. Although incorporating coronary angiography prior to radiotherapy planning may seem like the ideal option, it may not be feasible in most cases. Therefore, we began exploring the delineation guidelines for the LAD artery and came across those proposed by Lee et al. [8]. These guidelines are appropriate for use in clinical practice and aim to establish an anatomical boundary-based delineation definition for the LAD artery with adequate inclusion of the LAD artery and contouring consistency.

The International Quantitative Analysis of Normal Tissue Effects in the Clinic (QUANTEC) guidelines have subsequently been developed to predict the risk of cardiac mortality due to radiotherapy [15]. The QUANTEC guidelines state that, for partial heart irradiation, a V25 Gy <10% is associated with a <1% probability of cardiac mortality in long-term follow-up after radiotherapy. However, which region of the heart is functionally the most important for radiotherapy-induced cardiac toxicity remains uncertain. Previous studies have shown that mean heart dose (A4) is a better predictor of major coronary events than the mean dose to the LAD artery [5]. However, a previous study has shown an increase in high-grade coronary artery stenosis in the LAD artery of women who received left-sided radiotherapy for breast cancer, indicating a potential link between radiotherapy and coronary artery stenosis [13].

In our study, the mean doses of the LAD artery were significantly higher than that of the heart using both conformal techniques. Based on the pathophysiology, the LAD artery is the most important site for ischemic heart disease [8]. Evaluating radiotherapy plans by considering mean doses to the heart may drastically underestimate doses to the LAD artery, which may ultimately lead to long-term severe cardiac complications. In our study, among the substructures of the heart, doses to the LAD artery appear to be more clinically useful as a predictor of cardiac radiotoxicity.

In a study by Poitevin-Chacón et al. [16], the mean dose to the heart was 3.7 Gy, while that to the LAD artery varied from 3.66 to 53.01 Gy. In another study by Nilsson et al. [6], women with left-sided breast cancer had lower mean doses to the heart (range, 3-13 Gy) compared with mean doses to the LAD artery (range, from 18-55 Gy).

In our study, the mean dose to the heart was slightly lower (8.46 Gy in 3DCRT and 17.7 Gy in IMRT) than that to the LAD artery (23.66 Gy with 3DCRT and 31.53 Gy with IMRT). Tan et al. [17] reported very low mean (9.2 Gy) and maximum (24.6 Gy) doses to the LAD artery compared to the study by Poitevin-Chacón et al. [16] and our study. The studies show wide variations in the doses to the heart and LAD artery. Therefore, the mean dose to the heart may not be a good predictor of long-term cardiac morbidities.

A study by Jacob et al. [18] highlighted that the mean dose to the LAD artery (15.6 Gy) observed among 89 patients with left-sided breast cancer was substantially higher than that to the heart (2.9 Gy), which is similar to the findings of Drost et al. [19]. Our study also had similar results with the LAD artery receiving a higher dose than the heart with both techniques (23.66 and 8.46 Gy with 3DCRT and 31.53 Gy and 17.70 Gy with IMRT to the LAD artery and heart, respectively). This difference can be attributed to the fact that the LAD artery is located anterior to the heart and is more exposed to tangential fields than the entire heart.

Furthermore, Jacob et al. [18] emphasized that the mean heart doses are not representative of the order of magnitude of the mean dose to the LAD artery. The inter-individual variability in LAD artery exposure dose ranged from 1.6 to 37.6 Gy. The authors attributed the variation to several factors, such as variations in heart size, breast size, and individual coronary topology, and the presence or absence of regional lymph node variations.

As per QUANTEC, no dose recommendation exists for coronary arteries [20]. One Danish study [21] proposed a Dmax of 20 Gy to the LAD artery. In the present study, the Dmax to the LAD artery was 48.46 Gy; therefore, it is important to reduce the Dmax to the LAD artery. This has been discussed in a previous study by Poitevin-Chacón et al. [16]. Numerous strategies have been suggested to decrease the Dmax such as reducing the CT slice thickness (≤2.5 mm) using two tangential or multibeam IMRT plans, respiratory-gated radiotherapy, breath-holding techniques, and a prone patient position. Yeung et al. [22] found that, in patients with left-sided breast cancers, deep inspiration breath-hold was associated with a 43.5% reduction in the Dmean to the LAD artery compared to free-breathing radiotherapy. To decrease cardiac morbidity or mortality associated with radiotherapy, the Dmax to the LAD artery should be as low as feasible. Damage to any segment of the LAD artery can have devastating consequences; therefore, it is important to utilize different strategies to reduce the mean and maximal doses wherever possible.

We would like to suggest that long-term cardiotoxicity should be evaluated dosimetrically by assessing mean doses to the substructures of the heart rather than to the entire heart. This study showed a wide difference in doses between the entire heart and the LAD artery. A few other studies have also studied the mean doses to the left ventricle. Assessment of left ventricular function is based on echocardiography and correlates with symptoms, complications, and prognosis in several cardiac conditions.

In studies by Jacob et al. [18] and Mehta et al. [23], the mean dose to the ventricle was 6.2 Gy (higher than the mean heart dose of 2.9 Gy) and 13.69 Gy (higher than the mean heart dose of 8.48 Gy), respectively. Notably, the mean doses to the left ventricle were higher than those to the heart but lower than those to the LAD artery. It may be difficult to determine which substructure may be important for evaluating long-term cardiac morbidity. However, based on our results, we propose that the evaluation of cardiotoxicity based only on the mean heart doses requires further scrutiny. This study is unique in that it compared the dosimetric parameters of the heart and LAD artery along with the radiation techniques, namely, 3DCRT and IMRT.

Study limitations

This study had some limitations. First, the small sample size. This could be because the study analyzed data from a single center. Therefore, further studies with larger sample sizes are warranted. Second, this was a retrospective observational study. Therefore, causal relationships could not be established, and some unknown biases may have been introduced. Further prospective studies in this regard are warranted.

## Conclusions

There is a vast difference in the mean doses of the heart and LAD artery, and the mean doses of the heart as a long-term follow-up parameter for cardiac toxicity may be unreliable. In future studies, the mean doses to the cardiac substructures, especially to the LAD artery, should be evaluated, rather than those to the entire heart. Furthermore, long-term follow-up is needed to establish a dose-response relationship between radiation exposure and coronary artery stenosis.
